# Mental Health, Sleep Quality, and Psychological Well-Being during the Holy Month of Ramadan

**DOI:** 10.3390/healthcare12131301

**Published:** 2024-06-29

**Authors:** Danny Jandali, Abdullah Alwaleedi, Michele W. Marenus, Sarah R. Liener, Amine Sheik, Malak Elayyan, Weiyun Chen

**Affiliations:** 1 School of Kinesiology, University of Michigan, Ann Arbor, MI 48109, USA; djandali@umich.edu (D.J.); waleedi@umich.edu (A.A.); mmarenus@umich.edu (M.W.M.); sliener@umich.edu (S.R.L.); amines@umich.edu (A.S.); melayyan@umich.edu (M.E.); 2 College of Sports Science, University of Jeddah, Jeddah 23890, Saudi Arabia

**Keywords:** mental health, Ramadan, well-being, sleep quality, subjective happiness, life satisfaction

## Abstract

Objectives: Ramadan, a significant month for Muslims, presents unique challenges, particularly in the context of the USA. This study aimed to explore the relationship between mental health factors (depression, anxiety, and stress), sleep quality, and psychological well-being (subjective happiness and life satisfaction) during the month of Ramadan among participants and by gender. Methods: This study enlisted 163 participants (74% female, 25.7% male), with an average age of 36.8 years (SD = 13.1), mostly of Middle Eastern descent. Recruitment was conducted via flyers at local community mosques, social media, and outreach through local religious leaders. Data collection took place in the last three weeks of Ramadan, utilizing a Qualtrics survey that included the Depression Anxiety and Stress Scale (DASS-21), the Subjective Happiness Scale (SHS), Satisfaction with Life Scale (SWLS), and the Pittsburgh Sleep Quality Index (PSQI). Data were analyzed by means of descriptive statistics and multiple linear regression models using SPSS version 28. Results: The study indicates that while mental health and psychological well-being remained within normal levels during Ramadan, sleep scores indicated significant sleep disturbance among participants. Multiple linear regression models revealed that subjective happiness, sleep duration, and the global PSQI score were significant predictors of stress for the total sample (*F* = 9.816, *p* = 0.001). Life satisfaction was the only significant predictor of anxiety (*F* = 7.258, *p* = 0.001), and it, alongside subjective happiness, significantly predicted depression (*F* = 12.317, *p* = 0.001). For men, subjective happiness alone predicted stress, while life satisfaction was a predictor for both anxiety and depression (*F* = 4.637, *p* = 0.001). In women, sleep duration and medication usage were linked to stress but not anxiety. Life satisfaction and subjective happiness were, however, predictors of depression (*F* = 6.380, *p* = 0.001). Conclusion: Fostering positive affective states can serve as a protective mechanism against the potential psychological distress associated with altered sleep patterns and lifestyle changes that accompany Ramadan. This study highlights that Ramadan is a tool for bolstering happiness and life satisfaction, thereby lowering levels of stress, anxiety, and depression. In non-Muslim majority contexts like the USA, there is a need for accommodations to safeguard against potential psychological distress.

## 1. Introduction

The twenty-first century has witnessed heightened attention on the relationship between spirituality and religious factors affecting mental health and psychological well-being [1,2]. Religious components such as trust in God, reading and listening to the Quran, and participating in rituals like prayer and Ramadan fasting have been linked to decreased levels of depression, anxiety, and stress [2]. Ramadan, a pivotal month in the Islamic calendar, involves the practice of diurnal fasting observed by Muslims worldwide. This fasting period brings about substantial lifestyle adjustments as participants align their daily routines with the fasting regimen. Not only are individuals’ daily activities affected, but changes in their physical and mental performance, as well as their social life, are observed [3].

There is robust support for the positive effect Ramadan has on the mental well-being of the diverse populations who participate [2,4,5,6,7]. One review of 11 studies, which focused on the mental health of Iranian university students who engaged in Ramadan fasting, highlighted its profound influence on improving self-esteem and overall mental health [4]. These findings resonate with a study reporting an impressive reduction in anxiety and depression scores among over 80% of fasting individuals [5]. A similar study revealed an improvement in mental health scores among individuals who fasted the entire month of Ramadan. Conversely, lower scores of mental health were observed post Ramadan for groups who did not participate [6]. Moreover, a study of 182 elderly participants, who observed Ramadan fasting, exhibited significantly decreased levels of insomnia, anxiety, and depression. This shift in mental health is thought to be attributed to the heightened practice of spiritual beliefs and the presence of religious sentiments, giving individuals increased optimism and perceived control, thereby yielding greater life purpose [7].

It is crucial to recognize that the bulk of the existing literature on mental health and well-being during Ramadan originates from countries where Islam is the predominant cultural force. In these nations, Ramadan prompts extensive changes in daily routines and lifestyle practices, such as reduced work hours, increased social interactions, cultural celebrations, and altered meal schedules. The social atmosphere becomes distinctly more religious and spiritual. In contrast, Muslims in the United States face different circumstances, as the broader cultural and social adjustments seen in Muslim-majority countries are less pronounced or entirely absent. As a minority, Muslim-Americans may experience more challenges than benefits during Ramadan, as they must adapt to Western norms rather than having societal structures accommodate their fasting practices. Investigating these unique dynamics is essential to identify effective ways to better support fasting Muslims in the United States during Ramadan.

In addition, previous studies investigating mental health, sleep quality, and psychological well-being during Ramadan have been conducted on relatively small sample sizes or focusing on a singular sex (around 50 participants, predominantly male) [8]. In particular, the arrival of Ramadan often amplifies traditional Islamic gender roles, with women typically preparing evening meals while men tend to work. However, because Muslim women in the United States generally possess more autonomy and independence compared to those in some Islamic-majority nations, gender differences in mental health and psychological well-being are expected, giving way to identifying gender-specific accommodations down the line. Furthermore, few studies explore to what extent one’s level of sleep quality, happiness, and life satisfaction can predict one’s level of mental health, *during* the month of Ramadan. The majority of the literature has been focused on identifying changes in these parameters that come with participation in Ramadan, identifying differences in pre- and post-groups, without considering how these variables may influence each other during the month. To the best of our knowledge, there have been no previous studies conducted on this matter within the United States. Thus, the opportunity to explore the relationship between mental health, sleep quality, and psychological well-being during Ramadan within a context where such profound cultural shifts are absent remains unexplored.

Mental health disorders rank as one of the primary contributors to the worldwide burden of health issues, making research on this topic essential. Specifically, in 2019, depressive and anxiety disorders were among the top 25 causes of the burden worldwide, with no evidence of a reduction in the burden since 1990 [9,10]. The United States (US) stands out as one of the most affected nations concerning mental health issues. It is believed that one in five US adults are living with a form of mental illness. As of 2021, it is estimated that at least 21 million adults have experienced at least one major depressive episode in the last year [11]. Notably, since the onset of the COVID-19 pandemic, the Anxiety and Depression Association of America revealed that 7 out of 10 US adults experience daily stress or anxiety [12]. Furthermore, according to the National Institute of Health (NIH), rates of anxiety, depression, and substance use disorders have increased, and individuals with preexisting mental health conditions who contract the virus face a higher risk of mortality [13].

The World Health Organization refers to mental health as the “state of well-being in which the individual realizes his or her own abilities, can cope with the normal stresses of life, can work productively and fruitfully, and is able to make a contribution to his or her community” [14]. Under the umbrella of mental health, depression, anxiety, and stress are key components. Symptoms of depression involve a mental state characterized by persistent low mood, hopelessness, cognitive impairments, and behavioral issues, while symptoms of anxiety include excessive worry and physical symptoms such as restlessness and feeling on edge. Stress is operationalized as a state of emotional or mental strain, with feelings such as irritability, difficulty relaxing, and feeling overwhelmed [15]. Sleep quality has been defined as a construct consisting of both quantitative–objective and subjective measures of sleep [16]. Psychological well-being consists of two domains, of which the hedonic domain consists of life satisfaction, or the overall way one evaluates and perceives their life, and subjective happiness, largely measuring the ways an individual subjectively interacts with their world [17].

It is widely acknowledged that the components of mental health, such as stress, anxiety, and depression, are influenced by changes in the quality of sleep, life satisfaction, and subjective happiness [18,19,20,21,22]. Previous studies have investigated this relating to differences in gender and age. In a study of 331 male and female university students with an average age of 21.4 years, depressed individuals’ mean happiness and life satisfaction scores differed significantly compared to the scores of those without depression [18]. A study of 1293 college students with a mean age of 21.5 years reported that lower sleep quality significantly predicted an increase in depression and anxiety scores during the COVID-19 pandemic. Furthermore, the study found that females scored higher than males overall in levels of anxiety, depression, and poor sleep quality [19]. Previous studies have supported that components of mental health may be negatively affected by poor sleep [20,21]. Resonating with this, a study of 552 participants found that sleep quality and mental health parameters were related. Specifically, the Pittsburg Sleep Quality Index (PSQI) successfully predicted variances of 10.1%, 12.3%, and 13.1% in depression, anxiety, and stress, respectively [22].

Thus, the aim of this study is to investigate the extent to which levels of stress, anxiety, and depression are associated with levels of sleep quality, happiness, and life satisfaction during the month of Ramadan. This association was examined for the total sample, male sample, and female samples. It was hypothesized that levels of sleep quality, life satisfaction, and subjective happiness will be significantly and inversely associated with levels of stress, anxiety, and depression during the month of Ramadan across all three groups.

## 2. Methods

### 2.1. Participants and Setting

To maximize the representation of the target population, we sought to recruit as many participants as possible, ending with 163 total participants voluntarily taking part in this study. The mean age was 36.8 (SD = 13.1 years), and 57.7% of participants identified as Middle Eastern. The participants’ demographic information is displayed in Table 1. The participants were recruited in metro Detroit, the largest hub for Arab Americans in North America. Recruitment strategies included the distribution of flyers virtually and in person (handing out at local Muslim community centers), implementing a snowball recruitment method through word of mouth on social media and utilizing local religious leaders with access to large pools of potential participants. University clubs and organizations were also large targets for pools of participants. An incentive of a USD 25 Amazon gift card was raffled off to those who completed the survey. The study (HUM00226755) protocols were approved by the University Institutional Review Board (IRB) of Health and Behavioral Sciences.

### 2.2. Data Collection

A Qualtrics survey, containing four validated questionnaires, was distributed remotely to participants who met the inclusion criteria and consented to participating during the last three weeks of Ramadan. They were asked to complete the Qualtrics survey within a week of receiving it. Consent was obtained electrotonically prior to participants filling out the questionnaire. The consent form detailed the purpose of the study, the procedures involved, and confidentiality of their responses. Participants indicated their consent by selecting an option to agree before proceeding with the survey.

The Depression Anxiety and Stress Scale (DASS-21) designed by Lovibond and Lovibond [23] comprises three distinct sub-scales, each consisting of 7 items for 21 total items. Participants are asked to self-rate the presence of depressive, anxious, and stressful symptoms in the past week on a four-point Likert scale, ranging from 0 (did not apply to me) to 3 (applied most of the time). The aggregation of scores from the respective items, multiplied by two, will yield a participant’s total score, indicative of their level of depression, anxiety, and stress, with a higher score indicating higher levels. The seven-item stress sub-scale assessed difficulty relaxing, nervous arousal, being easily upset, agitated, irritable or overreactive, and impatience. The aggregated stress score ranges from 0 to 42. A score of 0–14 represents normal stress, 15–18 represents mild stress, and 27–34 represents severe stress. The seven-item anxiety sub-scale assessed autonomic arousal, skeletal muscle affects, situational anxiety, and subjective experience of anxious affect. The aggregated score ranges from 0 to 42. Normal anxiety scores fall in a range of 0–7, mild anxiety falls in the range of 8–9, and severe ranges from 15 to 19. The seven-item depression scale assessed dysphoria, hopelessness, devaluation of life, self-deprecation, lack of interest or involvement, anhedonia, and inertia. The aggregated score ranges from 0 to 42. A range of scores from 0 to 9 represent normal depression, scores ranging 10–13 represent mild depression, and scores from 21 to 27 represent severe depression. The DASS-21 has demonstrated its reliability and validity across genders in studies with large sample sizes (α = 0.95) [24].

The Pittsburg Sleep Quality Index (PSQI) was designed and validated by Buysse and his colleagues [25] to measure sleep quality in clinical populations. This self-rated questionnaire consists of 19 items, which generate seven component scores, each weighted equally on a 0–3 scale, with 0 representing no sleep difficulty, and 3 representing severe sleep difficulty. These seven components measure various attributes of one’s sleep. They include the following: (1) subjective sleep quality, (2) sleep latency, (3) sleep duration, (4) habitual sleep efficiency, (5) sleep disturbances, (6) use of sleeping medication, and (7) daytime dysfunction. The seven component scores are then summed to yield a global PQSI score, with a range of 0–21, with higher scores indicating worse quality of sleep. A total global PQSI sleep score of 0–4 indicates good sleep, while a sleep score of 5–21 indicates poor sleep. The PSQI has proved to be a reliable scale for measuring subjective sleep quality, with high test–retest reliability (r = 0.83) [26].

Satisfaction with Life Scale (SWLS), developed by Diener and his colleagues [27], was used to measure one’s overall life satisfaction. The scale consists of five items and uses a 7-point scale, ranging from 1 (strongly disagree) to 7 (strongly agree). Scores are then summed and assessed in a range of 5–35, where 5 represents extreme life dissatisfaction and 35 indicates extreme life satisfaction. The SWLS is reported to have strong internal consistency (α = 0.87) [28].

Subjective Happiness Scale (SHS), created by Lyubomirsky and Lepper [29], was used to assess an individual’s subjective happiness. The SHS contains a four-item questionnaire. Participant was asked to respond to each item with a 7-point rating scale, ranging from 1 (low happiness) to 7 (high happiness). The total score is calculated by summing the four items and dividing by four. The average score is 4.5–5.5, with scores below considered to represent lower-than-normal happiness and scores above 5.5 indicating higher-than-average happiness. The SHS offers insight into participants’ individual perceptions of happiness and has been shown to hold strong internal consistency across diverse samples (α = 0.76) [30].

### 2.3. Statistical Analysis

In this study, participants’ responses were gathered through structured questionnaires, and measuring these levels of mental health, sleep, happiness, and life satisfaction yielded numerical scores that fall along a continuum. Using these values will allow for the calculation of means and further correlational analysis for the total sample and male and female groups. Descriptive statistics of demographic variables and the study variables were conducted for the total sample, males only, and females only. Multicollinearity for each independent variable was tested using tolerance (T) and variance inflation factor (VIF). The results of T for all independent variables ranged from 0.610 to 0.924 (>0.01) and the values of VIF ranged from 1.082 to 1.640 (<5), an indication of no multicollinearity. Subsequently, standardized multiple linear regression coefficients were analyzed to assess the relative importance of each independent variable (sleep quality, life satisfaction, and subjective happiness) predicting each dependent variable (stress, anxiety, and depression) for the total sample, males only, and females only. All statistical analyses were performed using the SPSS statistical software (version 29.1, SPSS Inc., Chicago, IL, USA) with the significance level set at *p* ≤ 0.05

## 3. Results

### 3.1. Preliminary Results of the Study Variables

Table 2 presents the means and standard deviations of the depression, anxiety, and stress scores, and seven components of sleep quality, as well as life satisfaction and subjective happiness scores for the total sample, for males and females, respectively.

In this study, the mean scores of stress in all three groups fell within the range of normal stress (0–14). For the total sample, 128 (78.5%) participants showed normal levels of stress, 13 (8.1%) experienced mild stress, 9 (5.6%) were in moderate stress, 7 (4.3%) were in severe stress, and 4 (2.3%) had extremely severe levels of stress. With respect to anxiety, all groups mean scores were in the normal range (0–7), with females having the highest mean score. For the total sample, 109 (67.3%) experienced normal anxiety, 15 (9.3%) experienced mild anxiety, 23 (14.2%) were within moderate anxiety levels, 5 (3.1%) were in severe anxiety, and 10 (6.2%) were at extremely severe anxiety levels. Similarly, mean depression scores for the total sample, males, and females were within the range of normal depression (0–9), with males having higher depression on average. For the total sample, 121 (78.6%) participants experienced normal depression, 13 (8.4%) had mild levels of depression, 10 (6.5%) experienced moderate depression, 2 (1.3%) were in severe depression, while 8 (5.2%) were extremely severely depressed.

With regards to sleep quality, the total sample’s, males’, and females’ mean Global PSQI scores fell within the range indicating poor sleep (5–21). The total sample scored 6.48, females scored 6.42, and males scored 6.66. For sleep latency, the total sample scored 1.19, males scored 1.23, and females scored 1.18. For sleep duration, the total sample scored 0.89, while males scored 0.94 and females scored 0.87. Sleep disturbance scores for the total sample were 1.27, females scored 1.24, and males scored 1.37. Habitual sleep efficiency scores were among the lowest of the component sleep scores. The total sample scored 0.38, males scored 0.40, and females scored 0.37. The use of sleeping medication for the total sample was 0.38, for females 0.30, and for males 0.60. The daytime distinction score for the total sample was 1.15, for females 1.18, and for males 1.06.

With respect to life satisfaction, scores for the total sample, for males, and females all fell within a range of 21–25, representing moderate life satisfaction. For subjective happiness, the total sample and male and female groups scored similarly once again, falling in the range of normal happiness (4.5–5.5).

### 3.2. Association of Sleep Quality, Life Satisfaction, Subjective Happiness with Stress, Anxiety, and Depression—Total Sample

Table 3 presents the results of the multiple linear regression model with sleep quality and its components, life satisfaction, and subjective happiness predicting depression, anxiety, and stress for the total sample. The results indicated that the Global PSQI score and sleep components 1–6, life satisfaction, and subjective happiness were significantly associated with stress for the total sample (*F =* 9.816, *p <* 0.001), accounting for 41.4% of the total variance in stress scores. The standard regression coefficients reveal that subjective happiness (*β* = −1.818, *t* = −2.811, *p* = 0.006), sleep duration (*β* = −3.055, *t* = −2.813, *p* = 0.006), and Global PSQI score (*β* = 2.203, *t* = 2.480, *p* = 0.014) were individually significantly associated with stress. These results reveal that a higher level of subjective happiness and lower sleep duration is associated with decreased levels of stress, while a higher global sleep score is associated with an increased level of stress for the total sample.

In addition, the results indicated the Global PSQI score and sleep components 1, 3–7, life satisfaction, and subjective happiness were significantly associated with anxiety (*F =* 7.258, *p <* 0.001), accounting for 34.1% of the total variance in anxiety scores for the total sample. The results of the standard regression coefficients revealed that life satisfaction (*β* = −0.241, *t* = −2.358, *p* = 0.020) was the only individual significant contributor to anxiety scores. This result indicates that a higher level of life satisfaction is associated with a lower level of anxiety for the total sample.

In a similar vein, the results indicated that the Global PSQI score and sleep components 1–6, life satisfaction, and subjective happiness were significantly associated with depression (*F =* 12.317, *p <* 0.001), accounting for 48% of the total variance in depression scores. Subsequently, the standard regression coefficients revealed that life satisfaction (*β* = −0.365, *t* = −3.156, *p* = 0.002) and subjective happiness (*β* = −2.171, *t* = −3.701, *p* < 0.001) were individual significant contributors to depression. These results indicate that a higher level of life satisfaction and subjective happiness is associated with a decrease in depression scores for the total sample.

### 3.3. Association of Sleep Quality, Life Satisfaction, Subjective Happiness with Stress, Anxiety, and Depression—Male

Table 4 presents the results of the linear regression model with sleep quality and its components, life satisfaction, and subjective happiness predicting depression, anxiety, and stress for the male sample. The results supported that the Global PSQI score and sleep components 1–5, 7, life satisfaction, and subjective happiness were significantly associated with stress (*F =* 4.637, *p =* 0.001), accounting for 62.5% of the total variance in the male sample. The standard regression coefficients revealed that subjective happiness (*β* = −2.587, *t* = −2.494, *p* = 0.020) was the only individual significant contributor to stress for males. The results indicate that a higher level of subjective happiness is associated with decreased levels of stress in the male sample.

Furthermore, the results revealed that the Global PSQI score and sleep components 1–5, 7, life satisfaction, and subjective happiness were significantly associated with anxiety (*F =* 4.439, *p =* 0.001), accounting for 61.5% of the total variance in the male sample. The standard regression coefficients showed that life satisfaction (*β* = −0.346, *t* = −2.554, *p* = 0.017) and subjective sleep quality (*β* = 2.936, *t* = −1.209, *p* = 0.041) were individual significant contributors to anxiety in males. Thus, the results support that a higher level of life satisfaction is associated with lower levels of anxiety, and worse sleep quality is associated with increased levels of anxiety in the male sample.

In a similar light, the results indicated that the Global PSQI score and sleep components 1–5 and 7, life satisfaction, and subjective happiness were significantly associated with depression (*F =* 6.134, *p <* 0.001), accounting for 68.8% of the total variance in the sample. The standard regression coefficients revealed that life satisfaction (*β* = −0.514, *t* = −2.314, *p* = 0.029) was the single significant contributor to depression in males. These results indicate that a higher level of life satisfaction is associated with lower depression scores in males.

### 3.4. Association of Sleep Quality, Life Satisfaction, Subjective Happiness with Stress, Anxiety, and Depression—Female

Table 5 presents the results of the linear regression model with sleep quality and its components, life satisfaction, and subjective happiness predicting depression, anxiety, and stress for the female sample. The results supported that the Global PSQI score and sleep components 1, 3–7, life satisfaction, and subjective happiness were significantly associated with stress (*F =* 5.660, *p <* 0.001), accounting for 36.1% of the total variance in the female sample. The standard regression coefficients supported that sleep duration (*β* = −2.989, *t* = −0.2.713, *p* = 0.006), the use of a sleep medication (*β* = −2.770, *t* = −1.988 *p* = 0.050), and the global PSQI score (*β* = 2.149, *t* = −2.645, *p* = 0.010) were individual significant contributors to stress in females. These results support that lower sleep duration as well as increased use of sleeping medication is associated with decreased levels of stress, while a higher global PSQI score is associated with increased levels of stress for the female sample.

In addition, the results supported that the Global PSQI score and sleep components 1, 3–7, life satisfaction, and subjective happiness significantly predicted anxiety (*F =* 5.043, *p <* 0.001), accounting for 33.3% of the total variance in the female sample. The standard regression coefficients reveal that there were no significant individual contributors to anxiety for females.

Likewise, the results indicated that the Global PSQI score and sleep components 1, 3–7, life satisfaction, and subjective happiness were significantly associated with depression (*F =* 6.830, *p <* 0.001), accounting for 42% of the total variance in the female sample. The standard regression coefficients revealed that life satisfaction (*β* = −0.308, *t* = −2.168, *p* = 0.033) and subjective happiness (*β* = −2.065, *t* = −2.694, *p* = 0.008) were individual significant contributors to depression. Thus, these results support that a higher level of life satisfaction and subjective happiness is associated with lower scores of depression for the female sample.

## 4. Discussion

First, this study was pivotal in examining the levels of stress, anxiety, and depression as well as components of sleep quality, life satisfaction, and subjective happiness during Ramadan. Partially supporting the first hypothesis, the scores for mental health across the three groups indicate normal levels of depression, anxiety, and stress, which suggest the maintenance of a general level of mental well-being during Ramadan. This finding aligns with the broader narrative that spirituality and religious engagement, particularly during Ramadan, can contribute to the maintenance of mental health [2,4,5,6,7]. However, it is noteworthy that while participants reported satisfactory levels of mental health, life satisfaction, and subjective happiness, their Global PSQI fell within the range indicating poor sleep. This discrepancy between mental well-being and sleep quality could reflect the unique challenges—such as altered sleep schedules, strained work routines, and changing dietary habits—that individuals face during Ramadan, specifically within a nation where they may not be as accommodated. Given that Ramadan involves longer nightly prayers, poor sleep is often anticipated. Participants are advised to maintain a healthy sleep routine by engaging in physical activity in the evening after their daily meals. Furthermore, a review on the effect of Ramadan fasting on sleep duration and daytime sleepiness revealed a moderate reduction in total sleep time (TST) and a slight increase in daytime sleepiness scores during Ramadan [31], which echoes our findings of moderate daytime dysfunction and sleep disturbances across the three groups. These observations suggest that while fasting during this period can lead to decreased sleep duration, the impact on excessive daytime sleepiness is relatively minimal, indicating a nuanced effect of fasting on sleep quality and highlighting the need for further exploration. It is conceivable that participants’ sleep was affected by other factors such as a bodily change stemming from altered diet, altered medication routines, or smoking cessation. One aspect of sleep not considered is chronotype/circadian preferences. Individuals with a marked circadian preference for morning or evening hours might experience different sleep patterns and quality during Ramadan, which could influence the associations between sleep quality and mental health outcomes discussed in this study. Future research should evaluate these chronotype differences to better understand their impact on sleep quality and psychological well-being during Ramadan. Overall, it appears that the impact on sleep quality is counterbalanced by heightened spirituality and commitment to ritual practices, as evidenced by participants maintaining normal levels of mental health.

Furthermore, our study examined the associations between mental health indicators (stress, anxiety, and depression) and factors such as sleep quality, life satisfaction, and subjective happiness during the month of Ramadan. Consistent with our hypotheses, our study revealed significant associations between these variables for the total sample. Specifically, higher levels of life satisfaction and subjective happiness were associated with lower levels of depression, anxiety, and stress across the total sample. These findings corroborate previous research highlighting the protective role of positive affect and life satisfaction against psychological distress and mental health issues [18,32]. Additionally, poor sleep, as measured by the Global PSQI score, was linked to increased stress in the total sample and particularly among females, while in males, it appeared to contribute to heightened anxiety. This aligns with previous findings that suggest symptoms of poor mental health can be exacerbated by poor sleep quality [19,20,21,22]. Interestingly, although stress and anxiety were affected by poor sleep, there were no associations with indicators of depression. This observation prompts further investigation into whether participation in spiritual rituals such as Ramadan might buffer against the effects of poor sleep, a condition frequently reported during this period [3]. These findings underscore the crucial role of sleep quality as both a beneficiary and a detriment to mental health during Ramadan. Overall, our results suggest that Ramadan may serve as a mechanism for enhancing levels of subjective happiness and life satisfaction, which, in turn, contribute to reduced levels of stress, anxiety, and depression, as seen across our sample, while also mitigating the impact of poor sleep experienced during this period.

Although no statistical analysis between genders was performed, observations based on data shed light on gender differences. Females, on average, reported higher anxiety scores compared to males, while males reported higher levels of depression. This aligns with a 4-year longitudinal study that found females generally exhibited higher levels of general anxiety, whereas males experienced higher levels of depression [33]. These differences could result from varying societal expectations or gender roles associated with Islam. Women are often encouraged to express their emotions and assume household roles, which could lead to increased reports of anxiety and stress. Conversely, men may be discouraged from expressing emotions and are expected to act as a provider, potentially exacerbating feelings of isolation and depression. Interestingly, while sleep quality emerged as a significant predictor of mental health outcomes for the total sample, the associations varied between genders. Sleep quality did not appear to affect male stress levels, as their stress was solely influenced by subjective happiness. Conversely, factors of sleep quality, such as sleep latency, sleep duration, and the Global PSQI score, contributed to stress in females. This resonates with a study exploring gender differences in sleep, which analyzed 3778 young adults and indicated a higher prevalence of poor sleep quality in females compared to males (65.1% vs. 49.8%) [34]. For males, anxiety levels were exacerbated by poor subjective sleep quality and life satisfaction levels. On the other hand, females had no individual contributors for anxiety. These gender-specific differences underscore the complex interplay between sleep quality, life satisfaction, subjective happiness, and mental health outcomes, which may be influenced by sociocultural factors and individual coping mechanisms that vary between genders. Women in some cultures may not have as much autonomy or fewer opportunities to participate in social gatherings outside the home, impacting their sense of well-being during this month. Women also face unique health challenges during Ramadan, such as managing the month while menstruating, pregnant, or breastfeeding. These factors can affect their health differently from men. Moreover, it is possible intersectional differences play a role in the observed difference as one study on gender and caste differences in India reveals. Caste identities impact educational, occupational, and income choices for Muslim women. Upper-caste Muslim women face restrictions on their choices, whereas lower-caste women encounter severe marginalization and double discrimination. This dual oppression can significantly impact their mental health and well-being, as lower-caste women are often excluded from higher professions and forced into low-paid jobs, thereby exacerbating their sense of social and economic insecurity [35]. These insights suggest the need for tailored accommodations, such as workplace flexibility for males, or encouraging shared domestic responsibilities in households to alleviate the burden for females who are required to constantly prepare meals. In general, communities housing Muslim populations should strive for religious tolerance to better support those who partake in Ramadan. In environments where religious intolerance is prevalent, Muslim participants may face discrimination, marginalization, and lack of support for their religious practices. This can increase their stress levels and negatively affect their mental health during Ramadan.

This study has several limitations that warrant caution in interpretation and suggest directions for future research. First, its cross-sectional design limits the ability to draw causal conclusions about the relationships between the examined variables. Longitudinal studies are needed to better understand the temporal dynamics of these relationships, particularly how changes in mental health, sleep quality, life satisfaction, and subjective happiness evolve throughout the month of Ramadan and beyond. Instead of regression models, mediation/moderation models are recommended to allow for a better understanding of mechanisms linking these variables, instead of regression models. Second, the reliance on self-reported measures may introduce potential for response bias, where participants might overestimate positive behaviors or underreport negative symptoms. Future studies could benefit from incorporating objective measures of sleep quality, such as use of ActiGraph (Version 6.14.0) activity monitors, to provide a more accurate assessment. Third, the generalizability of findings may be limited by the sample’s cultural and religious composition. Research in more diverse contexts, including different non-Muslim-majority countries and among Muslims with varying levels of religious observance, would be valuable. Finally, the study’s focus on Ramadan within the USA might not fully capture the nuanced ways in which cultural, societal, and individual factors interact in other settings, suggesting the need for comparative cross-cultural research. Despite its limitations, including the cross-sectional design and reliance on self-reported measures, this research opens new avenues for tailored interventions aimed at enhancing well-being among fasting individuals.

## 5. Conclusions

This study sheds light on the psychological advantages of life satisfaction and subjective happiness in bolstering mental health amidst the distinctive challenges of fasting within a non-Muslim-majority setting. It also underscores the pivotal role of sleep quality as a potential factor influencing mental well-being. The findings imply that nurturing positive emotional states can function as a shield against potential psychological strain arising from altered sleep patterns and lifestyle adjustments during Ramadan. For practitioners and community leaders, these insights offer pathways for crafting tailored interventions aimed at enhancing well-being and mental resilience among fasting individuals in the United States. The observed gender differences underscore the necessity for gender-specific accommodations, advocating for the incorporation of mindfulness practices, community involvement initiatives, and well-being workshops that accentuate the spiritual and communal dimensions of Ramadan. Implementing such interventions could aid individuals in navigating the intricate balance between upholding traditional religious practices and assimilating into the broader societal fabric of a non-Muslim-majority country.

Overall, this study suggests avenues for future research to delve deeper into these dynamics, utilizing longitudinal methodologies or mediation/moderation models, as well as objective metrics to enrich our comprehension across diverse cultural and religious contexts. Such endeavors could contribute significantly to our understanding of the complex interplay between religious observance, psychological well-being, and sociocultural environments.

## Figures and Tables

**Table 1:** Participant demographics.

Variable		*n*	%
Gender	Female	121	74.2
	Male	42	25.8
Marital Status	Married	56	34.4
	Single	104	63.8
	Divorce	2	1.2
Education Status	High School Diploma	15	9.2
	Associate Degree	9	5.5
	Bachelor’s Degree	60	36.8
	Master’s Degree	45	27.6
	Doctoral Degree	34	20.9
Race	Asian	46	28.2
	Middle Eastern	94	57.7
	Black or African	6	3.7
	White	1	0.6
	Two or more	16	9.8

**Table 2:** Preliminary results of variables—descriptive statistics.

Variables	Total M (SD)	Female M (SD)	Male M (SD)
Stress	9.21(8.72)	9.34 (8.20)	8.85 (10.18)
Anxiety	7.02(6.56)	7.30 (6.95)	6.22 (5.26)
Depression	6.43 (8.24)	6.08 (7.71)	7.37 (9.58)
Satisfaction with Life	25.27 (5.89)	25.47 (5.90)	24.69 (5.90)
Subjective Happiness	5.11 (1.18)	5.12 (1.14)	5.05 (1.13)
Subjective Sleep Quality	1.22 (0.79)	1.28 (0.78)	1.06 (0.80)
Sleep Latency	1.19 (1.01)	1.18 (1.01)	1.23 (1.00)
Sleep Duration	0.89 (0.95)	0.87 (1.00)	0.94 (0.80)
Habitual Sleep Efficiency	0.38 (0.78)	0.37 (0.812)	0.40 (0.70)
Sleep Disturbances	1.27 (0.63)	1.24 (0.65)	1.37 (0.55)
Use of Sleeping Medication	0.38 (0.89)	0.30 (0.81)	0.60 (1.06)
Daytime Diysfunctionn	1.15 (0.85)	1.18 (0.87)	1.06 (0.80)
Global PSQI Score	6.48 (3.29)	6.42 (7.72)	6.66 (3.50)

**Table 3:** Association of stress, anxiety and depression with sleep quality, happiness, and life satisfaction—total sample.

Variable	R^2^	F	*p*	df	B	SE	*t*	*p*
Stress								
	0.414	9.816	<0.001	(125)				
Satisfaction with Life					−0.188	0.128	−1.469	0.114
Subjective Happiness					−1.818	0.647	−2.811	0.006
Subjective Sleep Quality					−0.165	1.579	−0.105	0.917
Sleep Latency					−0.140	1.126	−0.124	0.901
Sleep Duration					−3.055	1.086	−2.813	0.006
Habitual Sleep Efficiency					−1.524	1.110	−1.373	0.172
Sleep Disturbances					−2.749	1.498	−1.846	0.067
Use of Sleeping Medication					−2.491	1.264	−1.970	0.051
Global PSQI Score					2.203	0.88	2.48	0.014
Anxiety								
	0.341	7.248	<0.001	(126)				
Satisfaction with Life					−0.241	0.102	−2.358	0.020
Subjective Happiness					−0.557	0.512	−1.088	0.279
Subjective Sleep Quality					−0.635	1.038	−0.612	0.542
Sleep Latency					−0.117	0.756	−0.155	0.877
Sleep Duration					−1.183	0.960	−1.232	0.220
Habitual Sleep Efficiency					−0.761	1.125	0.677	0.500
Sleep Disturbances					−0.915	0.924	−0.990	0.324
Use of Sleeping Medication					.751	0.889	0.845	0.400
Global PSQI Score					.956	0.567	1.687	0.940
Depression								
	0.480	12.317	<0.001	(125)				
Satisfaction with Life					−0.365	0.116	−3.169	0.002
Subjective Happiness					−2.171	0.548	−3.811	0.001
Subjective Sleep Quality					−0.158	1.579	0.108	0.914
Sleep Latency					−0.211	1.126	−0.207	0.836
Sleep Duration					−1.565	1.086	−1.563	0.121
Habitual Sleep Efficiency					−0.792	1.004	−0.790	0.431
Sleep Disturbances					−1.500	1.349	−1.112	0.286
Use of Sleeping Medication					−0.769	1.151	−0.668	0.506
Global PSQI Score					−1.375	0.816	1.658	0.095

Note. Sleep component 7 (Daytime Dysfunction) was automatically excluded from the model as it was not a significant predictor of any outcome variables (stress, anxiety and depression).

**Table 4:** Association of stress, anxiety and depression with sleep quality, happiness, and life satisfaction—male sample.

Variable	R^2^	F	*p*	df	B	SE	*t*	*p*
Stress								
	0.625	4.637	<0.001	(25)				
Satisfaction with Life					−0.436	0.259	−1.684	0.105
Subjective Happiness					−2.857	1.146	−2.494	0.020
Subjective Sleep Quality					−4.046	2.607	1.552	0.133
Sleep Latency					4.636	2.810	1.650	0.111
Sleep Duration					1.060	2.435	0.435	0.667
Habitual Sleep Efficiency					2.933	2.691	1.090	0.286
Sleep Disturbances					1.559	2.971	0.525	0.604
Daytime Dysfunction					5.815	3.230	1.801	0.084
Global PSQI Score					−1.893	1.570	−1.205	0.239
Anxiety								
	0.615	4.439	0.001	(25)				
Satisfaction with Life					−0.346	0.136	−2.554	0.017
Subjective Happiness					−0.725	0.600	−1.209	0.238
Subjective Sleep Quality					2.936	1.365	2.151	0.041
Sleep Latency					0.796	1.471	0.541	0.593
Sleep Duration					−0.220	1.275	−0.173	0.864
Habitual Sleep Efficiency					0.338	1.409	0.240	0.812
Sleep Disturbances					1.073	1.556	0.690	0.497
Daytime Dysfunction					1.416	1.691	0.838	0.410
Global PSQI Score					−0.421	.822	−0.512	0.613
Depression								
	0.688	6.134	<0.001	(25)				
Satisfaction with Life					−0.514	0.222	−2.314	0.029
Subjective Happiness					−1.881	0.983	−1.913	0.067
Subjective Sleep Quality					2.708	2.238	1.210	0.237
Sleep Latency					0.081	2.412	0.034	0.973
Sleep Duration					−2.012	2.090	−0.962	0.345
Habitual Sleep Efficiency					1.272	2.310	0.551	0.587
Sleep Disturbances					−1.282	2.550	−0.503	0.619
Daytime Dysfunction					2.847	2.772	1.027	0.619
Global PSQI Score					0.511	1.348	0.379	0.708

Note. Sleep component 6 (Use of Sleep Medication) was automatically excluded from the model as it was not a significant predictor of any outcome variables (stress, anxiety and depression).

**Table 5:** Association of stress, anxiety and depression with sleep quality, happiness, and life satisfaction—female sample.

Variable	R^2^	F	*p*	df	B	SE	*t*	*p*
Stress								
	0.361	5.660	<0.001	(90)				
Satisfaction with Life					−0.134	0.155	−0.864	0.004
Subjective Happiness					−1.252	0.828	−1.513	0.390
Subjective Sleep Quality					−0.326	1.524	−0.214	0134
Sleep Duration					−2.989	1.072	−2.789	0.006
Habitual Sleep Efficiency					−1.185	1.323	−0.896	0.372
Sleep Disturbances					−2.675	1.619	−1.652	0.067
Use of Sleeping Medication					−2.770	1.393	−1.988	0.050
Daytime Dysfunction					−0.214	1.267	−0.169	0.866
Global PSQI Score					2.149	0.812	2.645	0.010
Anxiety								
	0.333	5.043	<0.001	(91)				
Satisfaction with Life					−0.258	0.134	−1.925	0.057
Subjective Happiness					−0.417	0.710	−0.587	0.559
Subjective Sleep Quality					−1.570	1.297	−1.210	0.229
Sleep Duration					0.246	0.926	0.265	0.792
Habitual Sleep Efficiency					−1.406	1.146	−1.227	0.223
Sleep Disturbances					1.026	1.401	0.732	0.446
Use of Sleeping Medication					−0.622	1.198	−0.519	0.605
Daytime Dysfunction					0.627	1.086	0.577	0.565
Global PSQI Score					1.127	0.703	1.604	0.112
Depression								
	0.420	6.830	<0.001	(85)				
Satisfaction with Life					−0.308	0.142	−2.168	0.033
Subjective Happiness					−2.065	0.766	−2.694	0.008
Subjective Sleep Quality					−0.490	1.413	−0.347	0.730
Sleep Duration					−1.408	0.992	−1.463	0.159
Habitual Sleep Efficiency					−0.618	1.212	−0.510	0.611
Sleep Disturbances					−1.336	1.485	−0.900	0.371
Use of Sleeping Medication					−1.414	1.274	−1.110	0.270
Daytime Dysfunction					−0.229	1.163	−0.197	0.845
Global PSQI Score					1.348	0.749	1.801	0.075

Note. Sleep component 2 (Sleep Latency) was automatically excluded from the model as it was not a significant predictor of any outcome variables (stress, anxiety and depression).

## Data Availability

The data presented in the study are available on request from the corresponding author.
